# Single molecule microscopy to profile the effect of zinc status on transcription factor dynamics

**DOI:** 10.1038/s41598-022-22634-x

**Published:** 2022-10-22

**Authors:** Leah J. Damon, Jesse Aaron, Amy E. Palmer

**Affiliations:** 1 Department of Biochemistry and BioFrontiers Institute, University of Colorado Boulder, Boulder, CO 80303 USA; 2 Advanced Imaging Center, Janelia Research Campus, Howard Hughes Medical Institute, Ashburn, VA 20147 USA

**Keywords:** Single-molecule biophysics, Metals

## Abstract

The regulation of transcription is a complex process that involves binding of transcription factors (TFs) to specific sequences, recruitment of cofactors and chromatin remodelers, assembly of the pre-initiation complex and recruitment of RNA polymerase II. Increasing evidence suggests that TFs are highly dynamic and interact only transiently with DNA. Single molecule microscopy techniques are powerful approaches for tracking individual TF molecules as they diffuse in the nucleus and interact with DNA. Here we employ multifocus microscopy and highly inclined laminated optical sheet microscopy to track TF dynamics in response to perturbations in labile zinc inside cells. We sought to define whether zinc-dependent TFs sense changes in the labile zinc pool by determining whether their dynamics and DNA binding can be modulated by zinc. We used fluorescently tagged versions of the glucocorticoid receptor (GR), with two C4 zinc finger domains, and CCCTC-binding factor (CTCF), with eleven C2H2 zinc finger domains. We found that GR was largely insensitive to perturbations of zinc, whereas CTCF was significantly affected by zinc depletion and its dwell time was affected by zinc elevation. These results indicate that at least some transcription factors are sensitive to zinc dynamics, revealing a potential new layer of transcriptional regulation.

## Introduction

Transcription factors (TFs) regulate gene expression through a tightly organized series of events, ultimately leading to the production of RNA by RNA Polymerase (RNAP). While all transcription requires a core group of general TFs to initiate transcription and guide RNAP to its target, many genes require additional cell-type specific TFs that bind to regions of DNA known as enhancers around the target gene to further stimulate or repress gene expression^1,2^. The activities of these TFs are tightly regulated by the cell. TFs are often inactive until they get activated by signals such as post-translational modification (e.g., phosphorylation) or translocation from the cytosol to the nucleus^3,4^. While much of the early work probing TF DNA binding properties was done in vitro, new technology has made it possible to probe these interactions in situ. One such technology is next-generation sequencing. Chromatin immunoprecipitation with sequencing (ChIP-seq) reveals with relatively good accuracy the sequences at which a given TF is bound. Additionally, computational techniques have been developed that use chromatin accessibility data from ATAC-seq to infer TF binding within regions of open chromatin^5,6^. However, these techniques have their drawbacks. First and foremost, these techniques only provide snapshots of what a particular TF is doing at a given treatment time. Additionally, most genomics assays are bulk assays that report averages from thousands of cells, which masks cell-to-cell heterogeneity^7,8^. Such heterogeneity can be critical to understanding diseases such as cancer.

Microscopy, namely single molecule (SM) microscopy, fills the single cell and single TF niche where genomics assays lag. While a previous barrier to studying TF activity was access to and expression of a fluorescent variant of the TF of interest, this has become much easier of late with the advent of overexpression systems such as the PiggyBac transposon system^9^ and the genome editing technique CRISPR^10,11^. Additionally, the development of robust protein labeling systems, such as HaloTag and its corresponding ligands^12,13^, has allowed researchers to expand into the SM field to monitor TFs in live cells. SM microscopy allows for evaluation of diffusion coefficients, search mechanisms, and dwell (binding) times for individual TFs, revealing intracellular and even intercellular differences amongst populations of TFs. This can also be paired with other imaging tools, such as those that study RNA production^14,15^, to further interrogate the functional consequences of TF activities.

Zinc finger TFs are among the most ubiquitous TF families in human cells, with nearly half (868/1792) of all predicted TFs utilizing zinc fingers to interact with genomic targets^16^. Although the precise coordination of the zinc ion (Zn^2+^) can vary, all of these TFs share the common attribute that the binding of Zn^2+^ stabilizes the proper fold of the zinc finger domain and thereby enables interaction with DNA^17^. It is well established that zinc finger TFs require their Zn^2+^ cofactor to bind DNA, but whether these TFs are sensitive to physiological changes in the labile Zn^2+^ pool in cells has not been examined. Multiple studies have shown that the labile Zn^2+^ pool in the cytosol and nucleus of mammalian cells is on the order of hundreds of picomolar^18–23^, but that the level of Zn^2+^ changes with an ever-expanding number of cellular processes. For example, it has been shown that cells experience changes in the labile Zn^2+^ pool during immune cell activation^24^, development^25,26^, neuronal stimulation^27^, and the cell cycle^19^, and that these fluctuations are important for cell physiology. Evaluating whether zinc-binding proteins, such as transcription factors, sense these fluctuations in Zn^2+^ is an important step in dissecting the mechanism of how Zn^2+^ fluctuations alter cell physiology.

The only known TF to exhibit sensitivity to the cellular Zn^2+^ pool is the metal-responsive transcription factor (MTF1). MTF1 senses high Zn^2+^ through its array of six zinc fingers and in the presence of high Zn^2+^, translocates to the nucleus to regulate metal buffering proteins (metallothionines) and metal transporters. The apparent dissociation constant (K_D_) for Zn^2+^ in the full length MTF1 protein was found to be 31 pM^28^. However, biochemical studies^29,30^ have shown that Zn^2+^ fingers five and six are the most reactive and have the weakest affinity for cobalt (which is often used to measure affinities of Zn^2+^ binding proteins^17^), suggesting that these may sense much higher levels of Zn^2+^. Most TFs that bind Zn^2+^ have K_D_s in the hundreds of picomolar range. For example, it’s been shown that the nuclear hormone receptors glucocorticoid receptor (GR) and estrogen receptor (ER) contain two C4 Zn^2+^ fingers bind Zn^2+^ with K_D_s of 316 and 501 pM, respectively^31^. Despite the fact that these K_D_s are similar to the concentration of the labile Zn^2+^ pool, these TFs are generally not thought to be metal responsive. However, this has not been tested in live cells.

In this work, we sought to determine whether zinc finger TFs are susceptible to changes in the labile Zn^2+^ pool. We used fluorescently tagged versions of GR, with two C4 Zn^2+^ finger domains, and CCCTC-binding factor (CTCF), with eleven C2H2 Zn^2+^ finger domains, and single molecule fluorescence microscopy to monitor their mobility within live cells. We found that CTCF, but not GR, shows increases in the mean squared displacement and apparent diffusion coefficient when cellular Zn^2+^ is chelated with Tris(2-pyridylmethyl)amine (TPA), suggesting that CTCF is more dynamic when cellular Zn^2+^ is low. Both Zn^2+^ and TPA decreased the dwell times for CTCF. On the other hand, GR was largely unaffected by perturbation of Zn^2+^. These results suggest that some TFs are sensitive to changes in the labile Zn^2+^ pool while others are not.

## Results

### CTCF, but not GR, shows significantly greater mean squared displacements and diffusion coefficient in low Zn^2+^

While it is well established that zinc finger transcription factors require Zn^2+^ to bind DNA in vitro, an open question is whether the Zn^2+^ occupancy and hence DNA binding capacity in cells is dependent on levels of cellular Zn^2+^. We applied 3D single molecule microscopy to investigate whether zinc finger transcription factors have altered mobility when Zn^2+^ is perturbed, where mobility is routinely used as a proxy for DNA binding^32–36^. Specifically, we used multifocus microscopy (MFM) that allows for simultaneous acquisition of particles in 9 Z-planes separated by approximately 430 nm (~ 3.9 µm total axial depth)^37^. This enabled us to track labeled transcription factors at rapid acquisition rates (25 Hz) and did not result in truncated trajectories as they diffused along the Z-axis in the nucleus, a common limitation of 2D single molecule tracking.

**Figure 1:**
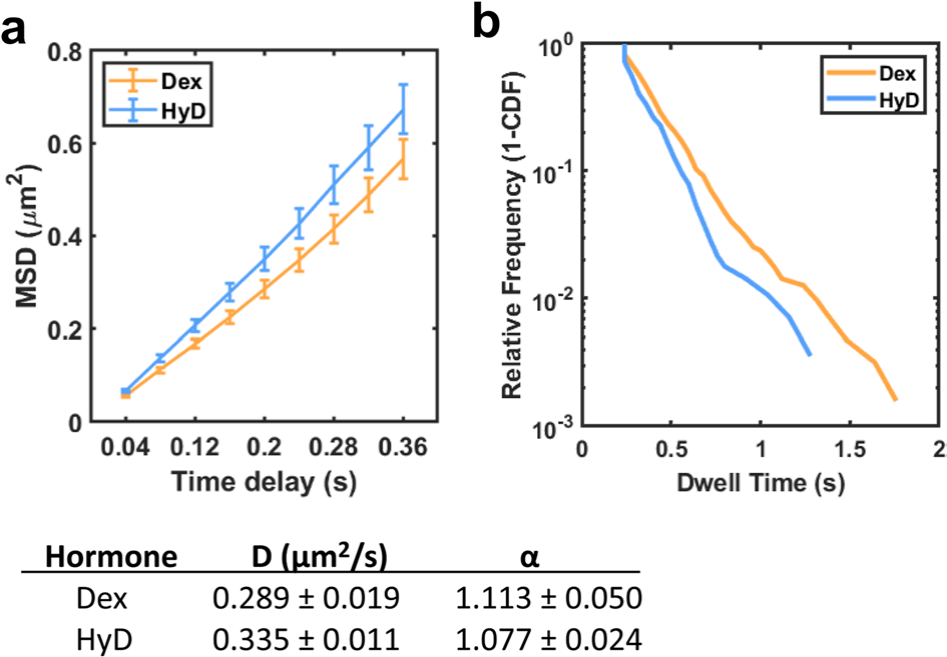
Dynamic properties of HaloTag-GR as measured by MFM. (**a**) Mean squared displacement (MSD) curves for HaloTag-GR treated with either 100 nM dexamethasone (Dex) or 100 nM hydrocortisone (HyD) for 30 min. Below, the calculated diffusion coefficients and α factors for each curve. (**b**) Dwell time analysis for HaloTag-GR treated with either 100 nM dexamethasone or 100 nM hydrocortisone. For both (**a**) and (**b**), a total of 236 and 151 trajectories were analyzed for dexamethasone and hydrocortisone treatment, respectively.

To test the ability of MFM to detect differences in TF dynamics, we examined the mean squared displacement and dwell times for the glucocorticoid receptor (GR) treated with the known activator dexamethasone or hydrocortisone. Previously it has been shown that dexamethasone is a more potent activator of GR than hydrocortisone and this leads to longer dwell times^34^, presumably because GR is more strongly associated with target sites on DNA. U2OS cells expressing HaloTag-GR (stable overexpression) were stained with JF549-HaloTag ligand and treated with 100 nM of either dexamethasone or hydrocortisone for 30 min. Figure 1a reports the mean squared displacements (MSDs) as a function of time for HaloTag-GR treated with each activator and the computed diffusion characteristics for each. GR activated by hydrocortisone diffuses more quickly than dexamethasone (diffusion coefficients of 0.335 ± 0.011 µm^2^/s and 0.289 ± 0.019 µm^2^/s for hydrocortisone and dexamethasone, respectively). These values are the diffusion coefficients for the entire population and most previous studies^36,38^ divide the populations into “fast” diffusing and “slow” diffusing populations. Therefore, we cannot directly compare these values to previous literature values. However, our observation that hydrocortisone results in more diffusive GR compared to dexamethasone is consistent with previous literature reports and confirms that our analysis is valid for analyzing TF dynamics. Additionally, we calculated the dwell (residence) times for GR treated with the above hormones. Our rationale was that particles with longer dwell times may correspond to those that are bound to DNA rather than freely diffusing or experiencing short, transient interactions^32–34,36,39^. We used the calculated MSDs from each trajectory to identify displacements of less than 200 nm that corresponded to bound particles. This 200 nm threshold has previously been used as a conservative estimate of general chromatin movement, as measured using fluorescently-labeled histone H2B^33,34^. Additionally, binding events had to last at least 8 frames (320 ms) to be considered bound; this eliminated transient, non-specific interactions that were sometimes observed. We found that hydrocortisone had slightly shorter dwell times than dexamethasone (Fig. 1b), consistent with our diffusion analysis and the model that GR activated by hydrocortisone would be bound to DNA for a shorter period of time than GR treated with the more potent activator dexamethasone. Taken together, these findings support that MFM is a valid technique for measuring differences in TF dynamics upon a chemical perturbation.

To evaluate whether perturbation of Zn^2+^ alters dynamics of candidate TFs, we continued to interrogate GR as it contains two C4 Zn^2+^ finger domains. Additionally, we selected the CCTCC-binding factor (CTCF), a chromatin binding protein that contains eleven C2H2 Zn^2+^ finger domains. Both transcription factors have been previously characterized by 2D single molecule microscopy and have DNA dwell times that span an approximate order of magnitude (3–8 s for GR^34^, 60 s for CTCF^32^). U2OS cells stably expressing HaloTag-GR or HaloTag-CTCF (CRISPR-edited endogenous expression) were treated with either 50 µM of the Zn^2+^ chelator TPA to deplete free Zn^2+^, 30 µM ZnCl_2_ to increase free Zn^2+^, or a media-only control for 30 min. Previously, we have shown that these perturbations decrease labile Zn^2+^ to < 1 pM or increase intracellular labile Zn^2+^ to 30 nM, respectively^19,20,40^. Under our imaging conditions, we acquired between 493–1080 total tracks for HaloTag-GR and between 5065–12,770 tracks for HaloTag-CTCF (Table 1), with most tracks lasting for less than 40 frames (1.6 s) (Supplemental Figure S1).

**Table 1:** Summary statistics for all cell lines and imaging conditions used in this study. Here, MFM denotes experiments conducted to generate 3D particle tracking data and N-STORM denotes experiments conducted to generate 2D particle tracking data.

Cell line	Treatment	Microscope	Exposure time (ms)	Acquisition interval (ms)	Number of cells	Total number of tracks	Total number of tracks – diffusion	Total number of Dwell times
U2OS HaloTag-GR	50 μM TPA	MFM	40	40	5	493	262	–
U2OS HaloTag-GR	Ctrl	MFM	40	40	6	948	419	–
U2OS HaloTag-GR	30 μM ZnCl_2_	MFM	40	40	5	1080	601	–
U2OS HaloTag-CTCF	50 μM TPA	MFM	40	40	11	11,704	5046	–
U2OS HaloTag-CTCF	Ctrl	MFM	40	40	10	12,770	4985	–
U2OS HaloTag-CTCF	30 μM ZnCl_2_	MFM	40	40	7	5065	1975	–
U2OS HaloTag-GR	50 μM TPA	N-STORM	100	100	8	17,511	N/A	7702
U2OS HaloTag-GR	Ctrl	N-STORM	100	100	11	45,748	N/A	10,994
U2OS HaloTag-GR	30 μM ZnCl_2_	N-STORM	100	100	9	24,544	N/A	20,121
U2OS HaloTag-CTCF	50 μM TPA	N-STORM	100	500	5	12,452	N/A	2721
U2OS HaloTag-CTCF	Ctrl	N-STORM	100	500	6	44,628	N/A	12,430
U2OS HaloTag-CTCF	30 μM ZnCl_2_	N-STORM	100	500	7	21,725	N/A	6179

**Figure 2:**
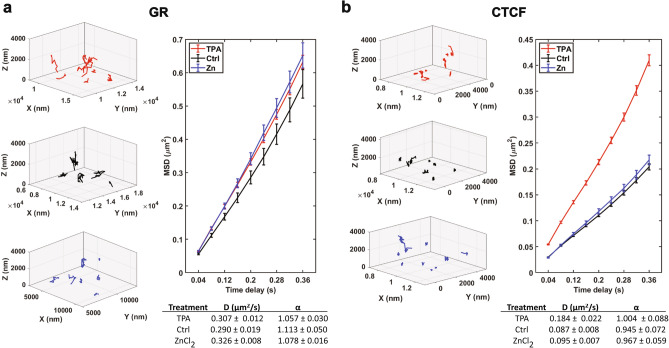
Dynamics of HaloTag-GR and HaloTag-CTCF upon perturbation of Zn^2+^, as measured by MFM. (**a**) Left, representative 3D SM trajectories for cells expressing HaloTag-GR treated with 50 µM TPA (red, top), a media-only control (black, middle), or 30 µM ZnCl_2_ (blue, bottom). Note that the volumes displayed for each treatment are equal, although the relative trajectory positions differ between conditions. Right, averaged mean squared displacement (MSD) curves as a function of time for each treatment, with the 95% confidence intervals for each time delay shown. Below, the calculated diffusion coefficients (µm^2^/s) and α values extracted from each curve are shown with 95% confidence intervals. (**b**) Same as in (**a**), but for cells expressing HaloTag-CTCF.

We observed that HaloTag-GR was much more diffusive (Fig. 2a, left) than HaloTag-CTCF (Fig. 2b, left). This observation is consistent with the TFs’ respective functions: GR is a canonical TF and diffuses throughout the nucleus to find its target genes while CTCF, as a chromatin architecture protein, forms more stable interactions with DNA. Upon perturbation of cellular Zn^2+^, we qualitatively observed that GR became slightly more diffusive whether Zn^2+^ was increased or depleted (Fig. 2a, left). We quantified this by calculating the mean squared displacements (MSDs) for each trajectory and plotting the aggregate MSDs for the first 9 displacements (corresponding to the first 10 frames of each trajectory, which was our minimum threshold for keeping high quality trajectories). From these MSD plots, we extracted the diffusion coefficients for the aggregate population of each treatment (Fig. 2a). We found that both increasing Zn^2+^ and decreasing Zn^2+^ resulted in slightly larger diffusion coefficients than the control cells (12.4% larger in high Zn^2+^, 5.8% larger in low Zn^2+^). A similar analysis for HaloTag-CTCF revealed that the MSDs upon Zn^2+^ depletion were significantly greater than either the control or Zn^2+^ treatment (Fig. 2b), resulting in a diffusion coefficient that was 211% higher than the control. Elevated Zn^2+^ resulted in a very small increase in the diffusion coefficient (9.1% compared to control). These results suggest that the dynamics of GR are minimally sensitive to changes in the labile Zn^2+^ pool, while CTCF is sensitive to Zn^2+^ depletion.

**Figure 3:**
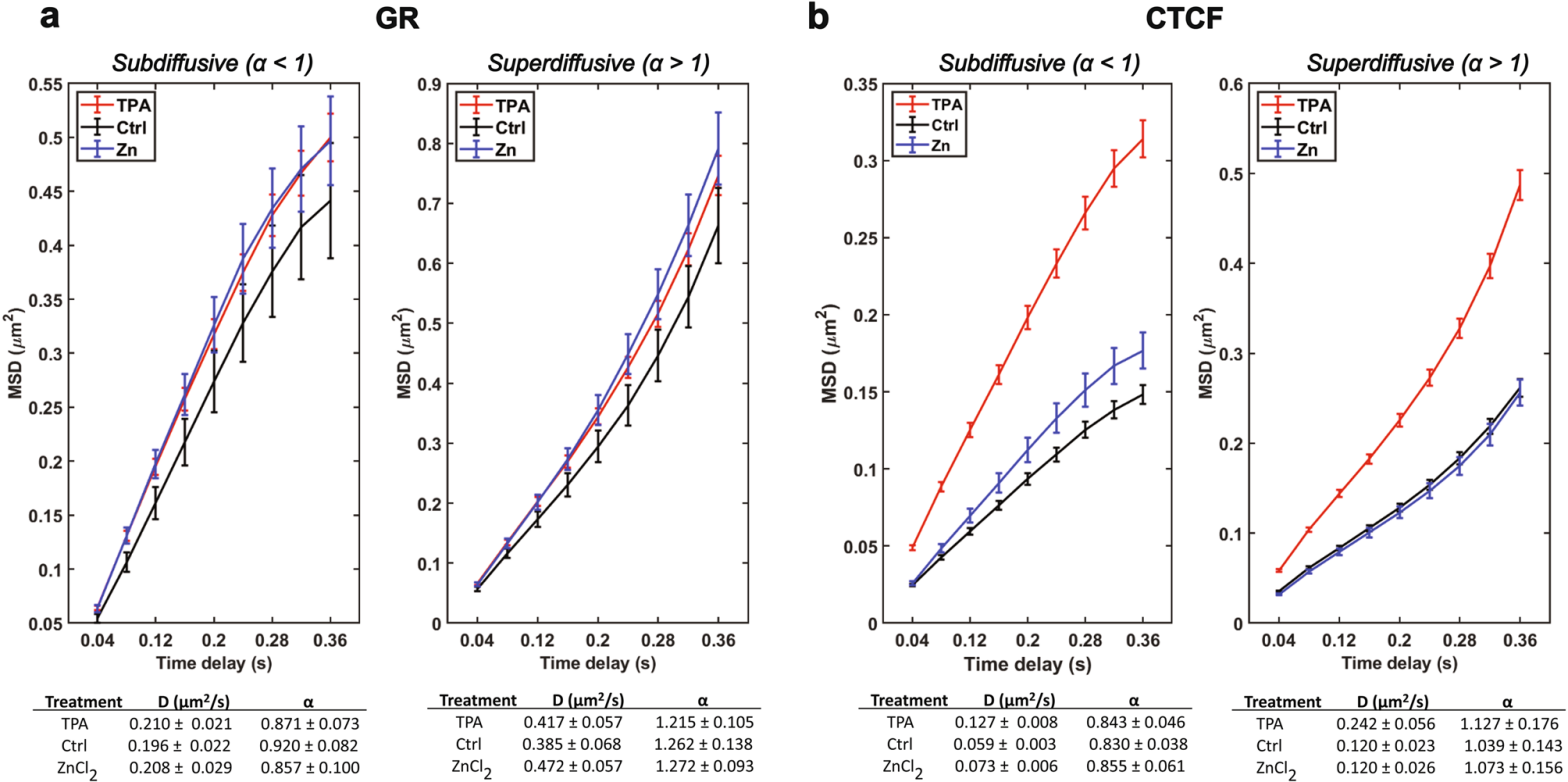
The effect of Zn^2+^ on subdiffusive and superdiffusive populations of HaloTag-GR or HaloTag-CTCF. (**a**) Subdiffusive and superdiffusive populations of HaloTag-GR expressing cells, treated with 50 µM TPA, 30 µM ZnCl_2_, or a media-only control. Lines indicate the respective population’s mean MSD at a given time delay, and error bars represent the standard error of the mean. Total number of superdiffusive trajectories: TPA, 653; Ctrl, 183; ZnCl_2_: 285. Total number of superdiffusive trajectories: TPA, 750; Ctrl, 236; ZnCl_2_: 316. (**b**) Same as in (**a**), but for cells expressing HaloTag-CTCF. Total number of superdiffusive trajectories: TPA, 2258; Ctrl, 2531; ZnCl_2_: 964. Total number of superdiffusive trajectories: TPA, 2788; Ctrl, 2454; ZnCl_2_: 1011.

The above analysis gives the diffusion coefficient for the entire population of molecules. However, proteins in cells are more apt to exhibit anomalous diffusion rather than purely Brownian motion, and sub-populations with different diffusion properties have been observed. The sub-populations of molecules with different diffusion behavior can be inferred by using the MSD curves to extract the $\alpha$ parameter. A value of $\alpha =1$ indicates purely Brownian motion, while $\alpha <1$ indicates subdiffusive behavior and $\alpha >1$ indicates superdiffusive behavior. Within our populations of HaloTag-GR and HaloTag-CTCF trajectories, we found that the aggregate $\alpha$ for the population hovered around 1 (Fig. 2). We separated these populations into those exhibiting subdiffusive or superdiffusive behaviors by fitting the individual MSD curve for each trajectory to the above diffusion model and extracted $\alpha$ values for each trajectory. Trajectories with $\alpha <1$ were labeled as subdiffusive and trajectories with $\alpha >1$ were labelled superdiffusive, and the diffusion coefficients were calculated for the two groups of molecules. For HaloTag-GR, there was a small increase in diffusion coefficient (D) for both subdiffusive (D was 7.1% larger in low Zn^2+^, 6.1% larger in high Zn^2+^) and superdiffusive particles (D was 8.3% larger in low Zn^2+^, 22.5% larger in high Zn^2+^) compared to control conditions (Fig. 3a). For HaloTag-CTCF, in low Zn^2+^ conditions there was a significant increase in D for both subdiffusive (215% larger compared to control) and superdiffusive particles (202% larger compared to control) (Fig. 3b). In high Zn^2+^ conditions, there was a small increase in D for subdiffusive particles (23.7% larger compared to control) and no change for superdiffusive particles.

### Dwell time analysis reveals that CTCF is more mobile in low Zn^2+^ conditions

As noted in Supplemental Figure S1, many of the MFM trajectories we detected lasted for less than 40 frames (1.6 s) due to photobleaching of the JF dye under the illumination conditions of the MFM. Literature reports for the dwell time of GR and CTCF are 7 seconds^36^ and 70 seconds^32^ respectively. Therefore, to determine whether the dwell times for GR and CTCF are altered by labile Zn^2+^, we performed a similar experiment using a Nikon N-STORM microscope in highly-inclined laminated optical sheet (HILO)^41^ mode with longer acquisition times and substantially less laser intensity. Using HILO we were able to image for 5 min at 10 Hz (for HaloTag-GR) and 20–30 min at 2 Hz (for HaloTag-CTCF) rather than the 1–2 min acquisition periods on the MFM. Additionally, these conditions allowed us to bias all detected particles towards bound particles, rather than freely diffusing particles. As such, we calculated the particle dwell time to be equal to $track length \times frame rate (s)$, with a minimum track length of 5 to be considered in the analysis.

**Figure 4:**
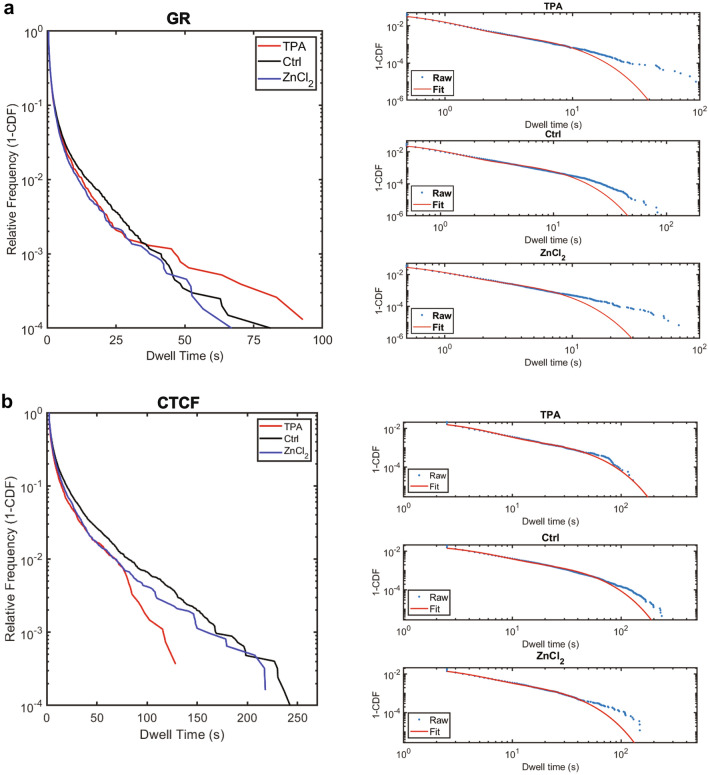
The effect of Zn^2+^ on dwell times, as measured by HILO**.** (**a**) Left: Raw 2D dwell time survival curves (1-CDF) for HaloTag-GR cells treated with 50 µM TPA (red), 30 µM ZnCl_2_ (blue), or a media-only control (black). Right: photobleaching-corrected survival curves for each treatment & their respective biexponential decay fits. (**b**) same as in (**a**), but with HaloTag-CTCF cells.

For HaloTag-GR, treatment with TPA and ZnCl_2_ resulted in very small changes in dwell times relative to the control (Fig. 4a). This correlates with the diffusion data we measured using the MFM. We first fit the survival curves to a biexponential decay (Fig. 4a, right) to extract the kinetic parameters ${k}_{1}$ and ${k}_{2}$, which correlate to the off rates for non-specific and specific interactions for the TF of interest. The inverse of ${k}_{2}$ ($\frac{1}{{k}_{2}})$ is widely used in the literature as the dwell time, $\tau$, of the TF. Through this method, we measured photobleaching corrected GR dwell times of 7.65 ± 0.35, 5.44 ± 0.38, and 3.94 ± 0.26 s for untreated, TPA-treated, and ZnCl_2_ treated cells (Table 2). These values are consistent with previously reported GR dwell times which range from 3–8 seconds^34,36^. Similarly, we find that perturbing Zn^2+^ with either TPA or ZnCl_2_ results in reduced dwell times for CTCF (Fig. 4b). This resulted in dwell times of 97.5 ± 11.03, 38.1 ± 4.43, and 30.1 ± 1.89 s for untreated, TPA-treated, and ZnCl_2_-treated cells, respectively (Table 2). Our measured dwell time for CTCF is slightly longer than the literature values (63–66 s)^32^. However, both TPA-treated and ZnCl_2_-treated cells exhibit dwell times substantially less than our untreated cells, indicating greater mobility of CTCF upon perturbation of Zn^2+^.

**Table 2:** Photobleaching-corrected 2D dwell time fits for HaloTag-GR and HaloTag-CTCF. ${k}_{1}$ and ${k}_{2}$ are the rate constants for the fast and slow components, respectively. $\tau$ is the dwell time calculated using ${k}_{2}$, and ${\tau }_{corrected}$ is the photobleaching corrected dwell time calculated using H2B dwell times calculated at the same frame rate.

Factor	Treatment	${k}_{1}$	${k}_{2}$	$\tau$(sec)	${\tau }_{corrected}$(sec)
HaloTag-GR	50 µM TPA	1.802 ± 0.044	0.237 ± 0.013	4.23 ± 0.23	5.44 ± 0.38
HaloTag-GR	Ctrl	1.655 ± 0.024	0.184 ± 0.006	5.44 ± 0.18	7.65 ± 0.35
HaloTag-GR	30 µM ZnCl_2_	1.802 ± 0.060	0.307 ± 0.017	3.26 ± 0.18	3.94 ± 0.26
HaloTag-CTCF	50 µM TPA	0.389 ± 0.016	0.056 ± 0.003	17.86 ± 0.98	38.1 ± 4.43
HaloTag-CTCF	Ctrl	0.302 ± 0.005	0.040 ± 0.001	25.00 ± 0.66	97.5 ± 11.0
HaloTag-CTCF	30 µM ZnCl_2_	0.400 ± 0.016	0.063 ± 0.002	15.87 ± 0.50	30.1 ± 1.89

**Figure 5:**
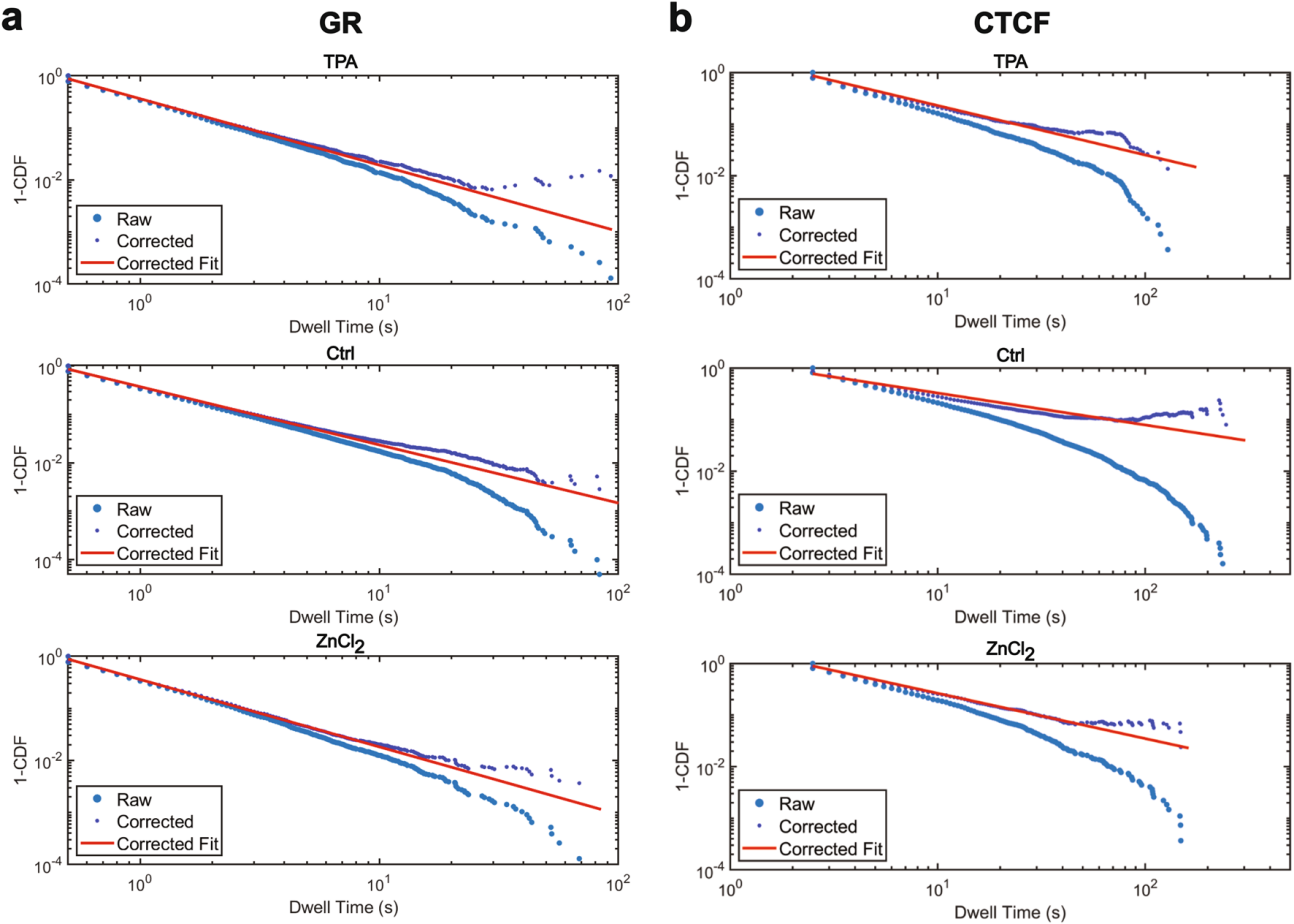
(**a**) Power law fits for HaloTag-GR for cells treated with 50 µM TPA (top), a media-only control (middle), or 30 µM ZnCl_2_ (bottom). Raw survival distributions were corrected for photobleaching using the survival curves from untreated U-2 OS cells stably expressing H2B-HaloTag. (**b**) Same as in (**a**), but for HaloTag-CTCF.

An alternative approach for comparing TF dwell times has recently emerged where the photobleaching-corrected dwell time survival curve (see methods) is fit to a power law ($f\left(t\right)=A{t}^{-\beta }$), rather than a biexponential decay^42^. The biexponential decay model assumes that a given TF occupies one of three states: diffusive; fast, or non-specific interactions; and slow, or specific interactions. This does not account for the transition from the fast state to the slow state, which a TF is likely to experience as it searches for its target site. When using a power law to fit dwell time survival curves, the physical model proposed is that of a broad distribution of affinities where there are microenvironments that contain energy wells of different depths. This model stems from the recognition that the chromatin landscape is heterogeneous due to physical constraints and motif degeneracy, and this heterogeneity affects TF binding. The power law exponent, $\beta$, is a measure of the skewness of the distribution where a smaller value of $\beta$ correlates with a longer dwell time. A previous study found that that $\beta$ values for GR and CTCF were 0.828 ± 0.004 and 0.55 ± 0.02, respectively^42^. When fitting our GR data to a power law, we found that the $\beta$ values were approximately 1.2 for all treatment conditions (Fig. 5a, Table 3). While these values of $\beta$ are larger than previously reported, they reveal no change in GR upon perturbation of Zn^2^^+^. When fitting our CTCF data to a power law, we found that the $\beta$ for untreated cells was 0.62 ± 0.03 (Fig. 5b, Table 3), which aligns closely with the previously reported $\beta$ of 0.55. We found that the $\beta$ for TPA treated cells was 0.95 ± 0.03 and the $\beta$ for ZnCl_2_ treated cells was 0.88 ± 0.02 (Fig. 5b, Table 3), indicating a decrease in DNA binding under these conditions.

**Table 3:** Power law fits for HaloTag-GR and HaloTag-CTCF. $\beta$ is a measure of the skewness of the distribution and is therefore proportional to dwell time.

Factor	Treatment	$A$	$\beta$
HaloTag-GR	50 µM TPA	0.36 ± 0.006	1.27 ± 0.03
HaloTag-GR	Ctrl	0.37 ± 0.005	1.20 ± 0.02
HaloTag-GR	30 µM ZnCl_2_	0.36 ± 0.006	1.29 ± 0.03
HaloTag-CTCF	50 µM TPA	2.08 ± 0.11	0.95 ± 0.03
HaloTag-CTCF	Ctrl	1.36 ± 0.03	0.62 ± 0.03
HaloTag-CTCF	30 µM ZnCl_2_	2.01 ± 0.09	0.88 ± 0.02

**Figure 6:**
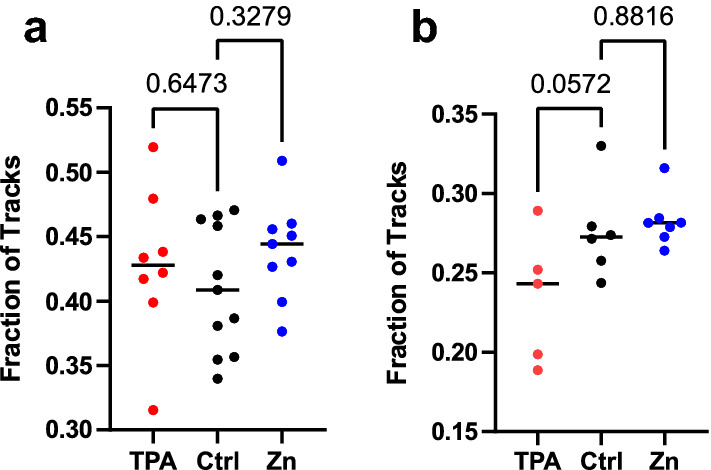
The effect of Zn^2+^ on fraction bound. (**a**) Fraction bound analysis for HaloTag-GR cells. Fraction bound was calculated as the number of tracks that were present for at least 5 frames (500 ms) divided by the total number of detected tracks. (**b**) Same as in (**a**), except with HaloTag-CTCF cells and with tracks that were present for at least 5 frames (2.5 s). Numbers indicate the p-value calculated using a one-way ANOVA.

Previous studies showed that deletion of the 11 Zn^2+^ fingers in CTCF resulted in long displacements consistent with free diffusion of CTCF^32^. This suggests that without Zn^2+^ fingers, CTCF is unable to effectively bind DNA. We set out to determine whether depletion of Zn^2+^ can alter the bound versus unbound trajectories for both HaloTag-GR and HaloTag-CTCF. The fraction bound was determined by dividing the number of trajectories longer than 5 frames by the total number of trajectories. While our imaging conditions did bias detections towards bound tracks, we only computed dwell times for trajectories lasting longer than 5 frames. For HaloTag-GR, we found that the fraction bound did not vary significantly across treatments (Fig. 6a). For HaloTag-CTCF, we found that the fraction bound for TPA treated cells was less than the control and ZnCl_2_ treated cells, with a p value = 0.0572 (Fig. 6b). We didn’t observe any difference between the cells treated with ZnCl_2_ and the untreated control.

## Discussion

Proper transcriptional regulation is essential for the cell’s ability to respond to the demands of its environment. Over the past several decades, various genomic techniques have been developed to monitor TF activity and the downstream consequences on gene expression, but most of these techniques monitor a heterogenous population of cells at fixed time points. SM microscopy allows for assessment of individual TFs within single cells, where different experimental approaches can be used to access different temporal regimes. For example, MFM is capable of rapid (> 25 Hz) acquisition in 3D due to simultaneous acquisition of multiple Z-planes. Thus, MFM is ideally suited for measuring fast TF characteristics such as diffusion coefficients. On the other hand, measurement of dwell times requires much longer measurement windows to observe the dynamics and heterogeneity of binding. HILO imaging is well-suited for such measurements and enables measurement of dwell times and fraction bound. Conveniently, many standard microscopes that are capable of total internal reflection fluorescent (TIRF) microscopy can be modulated to perform HILO imaging.

Bioinformatics studies predict that up to 10% of the human genome encodes for Zn^2+^ binding proteins, and nearly half (44%) are involved in transcriptional regulation^43,44^. While it is widely accepted that these proteins need their essential Zn^2+^ cofactor to function, very little is known about the metal status of these proteins in vivo, and whether these proteins are sensitive to changes in the labile Zn^2+^ pool. In mammalian cells, labile Zn^2+^ concentrations are in the hundreds of picomolar range^18–23,40^. While K_D_ values have only been measured for a small subset of zinc-binding proteins, the majority have K_D_ values in the low pM to nM range^17^, raising the possibility that some fraction of the zinc-proteome may be only partially saturated with Zn^2+^. There is increasing evidence that there are fluctuations in the labile Zn^2+^ pool that can range from low pM to nM during activation of immune cells^24^, development^26,45^, neuronal stimulation^23,27^, and the cell cycle^19^. Whether these fluctuations affect proteins in the zinc-proteome has not been examined.

In this work, we explored whether two Zn^2+^ dependent TFs sense perturbations in the labile Zn^2+^ pool. We found that HaloTag-CTCF was sensitive to decreases in the labile Zn^2+^ pool, where treatment with the Zn^2+^ chelator TPA led to increased mean squared displacement, an increase in the diffusion coefficient (> 200%), decreased dwell times, and a decrease in the fraction bound compared to control conditions. All of these results suggest that CTCF is more mobile in low Zn^2+^ conditions. The effect of elevated Zn^2+^ on CTCF was more subtle: there was a small increase in the diffusion coefficient (9.1%) and a decrease in the dwell time, but no change in the fraction bound. Perturbation of Zn^2+^ (either increase or decrease) had very little effect on GR; there were small changes in the mean squared displacement and diffusion coefficient (5–20% increase) and a slight decrease in dwell time for both treatments. These results suggest that GR is minimally affected by changes in the labile Zn^2+^ pool, while CTCF is primarily affected by decreases in Zn^2+^ and to a lesser extent by increases in Zn^2+^.

The differences in mobility, diffusion, and dwell time between GR and CTCF could result from changes in Zn^2+^ binding to the respective TFs upon Zn^2+^ perturbation, or could result from global changes in chromatin as a consequence of Zn^2+^ perturbation that affect CTCF more potently than GR. With respect to direct Zn^2+^ binding, the number and type of Zn^2+^ fingers used by these two proteins differ, with CTCF having 11 C2H2 Zn^2+^ fingers and GR having 2 C4 Zn^2+^ fingers. While the literature has shown that the mode of Zn^2+^ binding does not necessarily dictate the relative affinity of Zn^2+^ for isolated zinc  fingers^17^, most affinity measurements are carried out on individual zinc fingers and the binding properties could change in the context of the full length protein due to the larger second coordination sphere. CTCF has been shown to utilize different combinations of its Zn^2+^ fingers to bind distinct DNA sequences, allowing it to bind 80,000 + sites in the human genome^46^. While there is likely some redundancy in which of CTCF’s Zn^2+^ fingers are binding to DNA, it is plausible that perturbing the Zn^2+^ status of even one of these domains may affect the ability of CTCF to adequately bind its genomic targets. It is important to note that the dwell times we observe do not necessarily indicate functional binding events. As noted, CTCF can potentially bind 80,000 + sites in the genome, and GR ChIP-seq experiments have shown 10,000 + potential binding sites across multiple cell types^47^. However, of the ~ 7000 sites found in A549 (lung adenocarcinoma) cells, only 928 (13.5%) of these sites were shown to truly be glucocorticoid responsive. It is therefore plausible that some of the binding events we observed via single molecule microscopy may be dominated by transient, non-specific interactions that occur when a TF is searching for its target rather than specific, productive binding events. This is where novel quantitative methods and kinetic models for assessing TF dwell time, such as fitting distributions to a power law to account for a continuum of binding affinities^42^, are needed to deconvolve the complexity of transcriptional regulation. With respect to global changes in chromatin, we cannot rule out the possibility that perturbation of Zn^2+^ leads to changes in chromatin architecture. Besides TFs, Zn^2+^ is a required cofactor for histone acetyltransferases, histone deacetylases, histone demethylases, and DNA methyltransferases^48^. It may be that the sheer number of Zn^2+^ binding proteins in the nucleus all contribute to changes in chromatin accessibility or compaction that alter the dynamics of TFs. CTCF, as one of the key chromatin architecture proteins, may therefore be more strongly affected by perturbations in Zn^2+^ than GR, perhaps because of the number of genomic binding sites. Whether the effect of Zn^2+^ is direct (modulating the Zn^2+^ occupancy of the TF of interest) or indirect (changing chromatin architecture in way that affects some TFs more than others), the implication of our results is that some TFs are affected by physiological changes in the labile Zn^2+^ pool and hence their function could be altered during Zn^2+^ fluctuations.

Our work suggests that a subset of TFs may be sensitive to changes in cellular Zn^2+^. While further studies are necessary to determine whether changes in TF dynamics and mobility are correlated with altered function, our results show for the first time that canonical zinc-finger TFs (outside of MTF1) can be sensitive to changes in the labile Zn^2+^ pool. Given the noted advantages of SM microscopy, it would be valuable to pair these techniques with other fluorescent microscopy techniques to assess the downstream consequences of this sensing. GR, as a canonical TF, could be paired with the incorporation of promoter arrays to more readily assess specific binding^34^, or it could be coupled with nascent RNA imaging^14,15^ to examine the consequences on its target gene products. CTCF, as a regulator of chromatin architecture, could be paired with microscopy techniques that measure chromatin compaction^49–51^ to see if this is perturbed with changes in Zn^2+^. These tools will better allow us to understand precisely how changes in TF dynamics translate into changes in TF function.

## Materials and methods

### Plasmid generation

To generate PiggyBac-GR-HaloTag, PB-CMV-MCS-EF1α-Puro (System Bioscience #PB510B-1) was linearized using EcoRI and BamHI. The GR insert was amplified from pk7-GR-GFP (Addgene #15534) to generate overhangs with both PB-CMV-MCS-EF1α-Puro and the HaloTag. The HaloTag insert was amplified from pcDNA3.1-3xFLAG-HaloTag-2xNLS (Daniel Youmans, Cech lab, CU Boulder) using the primers listed in the Key Resources table to generate overhangs with GR and PB-CMV-MCS-EF1α-Puro. Linear fragments were then assembled into the final plasmid using a homemade Gibson assembly master mix.^52^.

### Cell culture conditions

All cells were cultured in Dulbecco’s Modified Eagle Medium (DMEM) (ThermoFisher #12800082) containing 10% FBS (SigmaAldrich #F0926)) and 1% penicillin/streptomycin (ThermoFisher #15140–200). All live cell single molecule tracking experiments were carried out in FluoroBrite DMEM (ThermoFisher #A1896701). To generate stable U-2 OS HaloTag-GR and H2B-HaloTag cells cells, wildtype U-2 OS cells (ATCC #HTB-96) were transfected with 1 µg of either PiggyBac-HaloTag GR or PiggyBac-H2B-HaloTag (Luke Lavis, HHMI Janelia), 250 µg of Super PiggyBac Transposase (System BioSciences #PB200A-1), and 3 µL of TransIt LT1 (Mirus #MIR2305). Stable clones were selected by growing in DMEM containing 0.5 µg/µL puromycin (Sigma-Aldrich #P8833-25MG) for 7 days, after which they were transferred to normal DMEM. Cells expressing endogenous HaloTag-CTCF were a gift from Anders Serj Hansen (MIT)^32^.

### Zinc perturbations

To manipulate labile Zn^2+^, cells were treated with either 30 µM ZnCl_2_ (Sigma-Aldrich # 39059-100ML-F) or 50 µM Tris(2-pyridylmethyl)amine (TPA, Sigma-Aldrich # 723134-250MG) for 30 min prior to imaging. For experiments involving HaloTag-GR, Zn^2+^ perturbations were followed by hormone activation with either 100 nM dexamethasone (Sigma-Aldrich #D4902-100MG) or 100 nM hydrocortisone (Sigma-Aldrich #H4001-1G).

### 3D single particle tracking

Three dimensional single particle tracking was performed on the Multifocus Microscope (MFM) at the Janelia Advanced Imaging Center (HHMI)^37^. Briefly, an epi-fluorescent microscope equipped with a multi-focal diffraction grating (MFG) allows for the collection of 9 aberration-corrected focal planes. Chromatic aberrations are corrected using a separate chromatic correction grating and prism. The MFG allows for a total axial detection depth of approximately 4 µm. For each experimental day, a calibration with TetraSpeck 0.2 um fluorescent beads (ThermoFisher #T7279) was used to determine the precise Z-spacing between each focal plane, with an average ΔZ of 430 nm. Additionally, the calibration allowed for measurement of the point spread function of the microscope, which allowed for image deconvolution (see below). All images were acquired using a 100 × 1.45 NA TIRF objective (Nikon), a 561 nm laser (Cobolt Jive 300, ), a Di01-R405/488/561/635 dichroic (Semrock), a FF01-593/40 (Semrock) emission filter, and an iXon3-DU897E EMCCD (Andor Technologies).

*Image acquisition:* Cells were labeled with 1 µM of JaneliaFluor (JF) 549 HaloTag ligand (Janelia Research Campus) for 5 min at 37 °C, rinsed three times with Dulbecco's phosphate-buffered saline (D-PBS), and then incubated for 30 min in FluoroBrite DMEM. Image acquisition was performed using a 561 nm laser at typical irradiance of 3-4 kW/cm^2^, with 40 ms exposure times for an effective frame rate of 25 Hz. Movies, on average, were acquired for 2 min (3000 frames).

*Post processing:* Following acquisition, movies were cropped and laterally registered using a pre-determined affine transformation determined via TetraSpeck bead data (described above) to convert the 3 × 3 image into a 9 Z-plane stack, spanning approximately 3.8 µm in the Z-dimension. To improve signal-to-noise ratio and particle localization, local background was subtracted using a rolling ball correction (radius = 7 px), followed by 5 iterations of the Richardson-Lucy deconvolution algorithm within MATLAB R2019a (Mathworks). Following deconvolution, images were smoothed using a Gaussian filter (radius = 0.7 px) to improve particle detection. Additionally, the first 500 frames of each movie were removed, as these tended to have dense labeling that did not allow for robust tracking.

*3D particle tracking*: Particle trajectories were generated using the MosaicSuite ImageJ plugin^53^ with the following parameters: radius = 3 px; cutoff = 0.001; threshold = 750; max link range = 1 frame; max displacement = 500 nm; dynamics = Brownian. Additionally, any tracks that did not exist for at least 10 frames (400 ms) were discarded.

*Diffusion analysis:* Diffusion coefficients for HaloTag-GR and HaloTag-CTCF were calculated by first computing the mean squared displacement (MSD) for each trajectory across the entire length of the track. The first 9 displacements (corresponding to the first 10 frames of each trajectory) of all tracks in each condition were subsequently averaged to generate an aggregate MSD curve across the population. This curve was then fit to the equation $MSD=\gamma D\Delta {t}^{\alpha }$, where *MSD* is the mean squared displacement, $\gamma$ is the number of dimensions (3) multiplied by 2, $D$ is the apparent diffusion coefficient, $\Delta t$ is the time delay between frames (here, 0.040 s), and $\alpha$ defines whether the population exhibits superdiffusive ($\alpha >1$) or subdiffusive ($\alpha <1$) behavior. In addition to calculating the diffusion coefficients for each population, we further divided the population into trajectories exhibiting superdiffusive and subdiffusive behaviors by fitting the MSD curves of individual trajectories to the above equation and filtering according to the $\alpha$ measured for each.

### 2D single particle tracking

*Equipment:* 2D single particle images were acquired on a Nikon N-STORM imaging system equipped with a Nikon TI-E microscope, a Nikon CFI Apo TIRF 100X oil immersion objective (1.49 NA), a N-STORM 647 nm laser (Agilent), an iXon 897 Ultra EMCCD (Andor Technologies), and a cage incubator (Okolab).

*Image acquisition*: Cells were stained with JaneliaFluor (JF) 646 HaloTag ligand at either 100 pM (U-2 OS HaloTag-CTCF) for 1 min or 1 nM (U-2 OS HaloTag-GR) for 5 min, rinsed three times with D-PBS, and then incubated for 30 min in FluoroBrite DMEM prior to Zn^2+^ perturbations. Image acquisition occurred with low laser intensities (5–10%) and 100 ms exposure times. Frame rates were chosen to bias towards only detecting bound particles: for HaloTag-CTCF, effective frame rate = 2 Hz; for HaloTag-GR, effective frame rate = 10 Hz. Because CTCF is known to have long residence times on DNA, these movies were collected on average for 20 min (2400 frames), while movies for GR were typically acquired for 2–5 min.

*Post processing:* Movies were post-processed within Nikon Elements to subtract background using the rolling ball method (radius = 50), and then subjected to 5 iterations of the Richardson-Lucy deconvolution algorithm within Nikon Elements. Following deconvolution, images were smoothed using a Gaussian filter (radius = 0.7 px) to improve particle detection. Additionally, the first 500 frames of each movie were excluded, as these tended to have dense labeling that did not allow for robust tracking.

*2D particle tracking*: Particle trajectories were generated using the MosaicSuite ImageJ plugin with the following parameters: radius = 3 px; cutoff = 0; absolute threshold = 500–1000, depending on the experiment; max link range = 1 frame; max displacement = 300 nm; dynamics = Brownian. Additionally, any tracks that did not exist for at least 5 frames (2.5 s for CTCF and H2B; 0.5 s for HaloTag-GR) were excluded from further analysis.

*Dwell time analysis:* Because the effective frame rate of each movie and tracking parameters were biased towards only detecting bound molecules, we inferred that the only particles detected were bound. Therefore, we calculated dwell time as the length of the track divided by the frame rate, and the aggregate dwell times were used to generate dwell time survival curves. These were then fit to a biexponential decay $f\left(t\right)=a{e}^{({k}_{1}t)}+ b{e}^{({k}_{2}t)}$, where $a$ and $b$ are the fraction sizes of the two components and ${k}_{1}$ and ${k}_{2}$ are the off rates for non-specific and specific binding. For the traditional photobleaching correction of these data, we assumed that the calculated value ${k}_{2}$ is skewed due to photobleaching as previously noted^32^. Therefore, we used H2B dwell time data acquired at the same frame rate (10 Hz for GR, 2 Hz for CTCF; Supplemental Figure S2) to estimate ${k}_{bias}$, where ${k}_{bias}={k}_{2,H2B}$. This then enabled us to calculate ${k}_{2, true}= {k}_{2}-{k}_{bias}$. Dwell times for each component were subsequently computed as ${\tau }_{corrected}= \frac{1}{{k}_{2, true}}$.

For fitting the dwell times to a power law, photobleaching was first corrected using H2B-HaloTag as previously described^42^. Briefly, the raw H2B-HaloTag dwell time survival curves were fit to a triple exponential of the form:

$$P\left({\tau }_{his} \ge t\right)={f}_{1}{e}^{-{\gamma }_{1}t}+{f}_{2}{e}^{-{\gamma }_{2}t}+ {f}_{3}{e}^{-{\gamma }_{3}t}$$

Here, ${\gamma }_{1}$ and ${\gamma }_{2}$ are related to the dynamics of histones, but ${\gamma }_{3}$ corresponds to the rate of photobleaching. This subsequently allowed us to correct the survival distribution for a TF of interest, $P\left({\tau }_{TF} > t\right)$, by calculating:

$$P\left({\tau }_{TF, real} \ge t\right)=\frac{P({\tau }_{TF}\ge t)}{{e}^{-{\gamma }_{3}t}}$$

The survival distribution was then fit to a Power law, $f\left(t\right)=A{t}^{-\beta }$, where $\beta$ is proportional to the dwell time of the TF.

## Supplementary Information

Supplementary Information.

## Data Availability

The datasets generated and analyzed during the current study are not publicly available due the large size of the time-lapse imaging data but are available from the corresponding author on request.
