# Supplementary material for: Single molecule microscopy to profile the effect of zinc status on transcription factor dynamics
Source: Sci Rep. 2022 Oct 22;12:17789. doi: 10.1038/s41598-022-22634-x (PMC9588069; doi:10.1038/s41598-022-22634-x)
Supplement: Supplementary file 1 — Supplementary Information. [file 41598_2022_22634_MOESM1_ESM.pdf]

Supporting Information for:

Single molecule microscopy to profile the effect of zinc status of transcription factor dynamics

Leah J. Damon[1], Jesse Aaron[2], Amy E. Palmer[1*]

Supporting Figure S1: Trajectory length probability density distributions for all 3D particle tracking data.

Supporting Figure S2: H2B dwell times for U-2 OS cells stably expressing H2B-HaloTag.

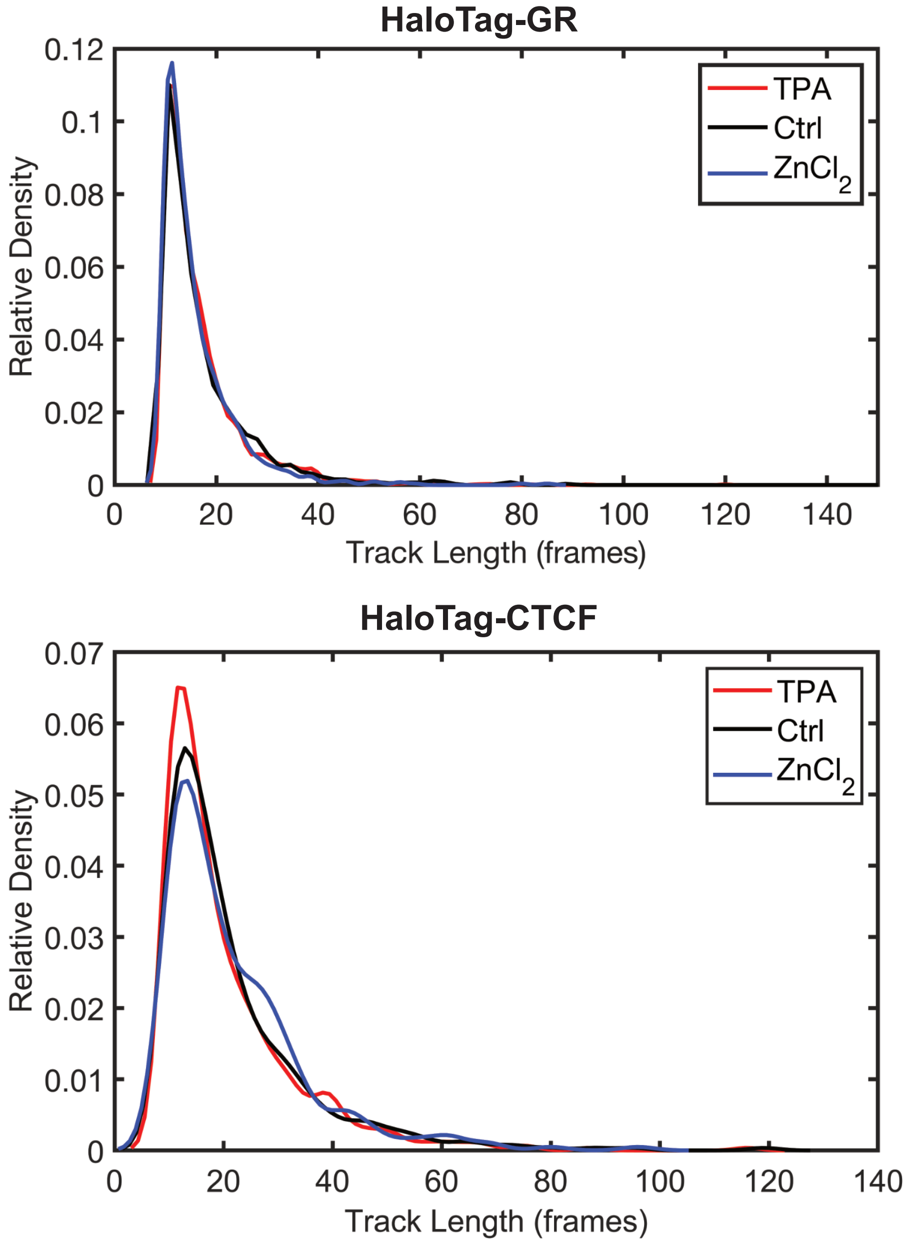

Supplemental Figure S1. Trajectory length probability density distributions for all 3D particle tracking data. For each cell line and treatment, trajectory lengths from each replicate were pooled and the probability density distributions were generated using the *ksdensity* function in MATLAB (R2020a). Total numbers of trajectories for each condition can be found in Table 1.

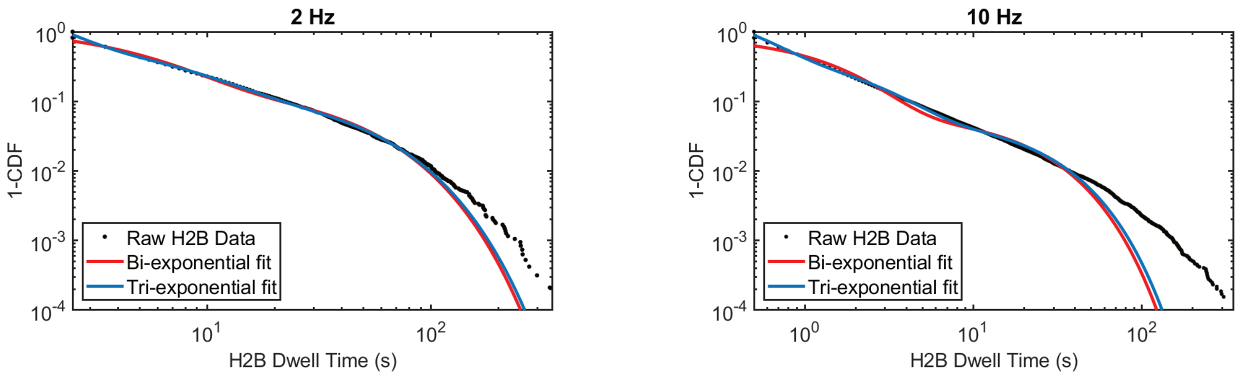

Supplemental Figure S2. H2B dwell times for U-2 OS cells stably expressing H2B-HaloTag acquired at 2 Hz (left, corresponding with HaloTag-CTCF acquisition conditions) and 10 Hz (right, corresponding with HaloTag-GR acquisitions). Raw dwell time survival curves were fit to either a biexponential or a triexponential decay to correct HaloTag-GR and HaloTag-CTCF dwell time data for photobleaching.
